# General Reports of the Royal Hospitals of Bridewell and Bethlem, for the Years Ending 31st December 1843 and 1844

**Published:** 1845-10

**Authors:** 


### Art. XVII.

*General Reports of the Royal Hospitals of Bridewell and Bethlem, for the Years ending* 31*st December* 1843 *and* 1844. Printed for the use of the Governors.—*London,* 1844-5. 8vo, pp. 82-112.

The Royal Hospital of Bethlem, commonly called "Bedlam" is a government institution containing three classes of patients.

Poor lunatics are admitted who have not been insane more than twelve months, and they are allowed to remain twelve months under cure; some of these remain as incurables; and the third class consists of criminal lunatics. In 1844 there were 182 curable cases, 86 incurables, and 91 criminal lunatics. The Reports of 1843-4 are now before us: important documents as careful registers of facts, and from the proofs they exhibit of the judicious, watchful, and humane treatment adopted. They breathe throughout the spirit of humanity and good sense, and convey the impression that the committee and staff perform their duties admirably well. We shall be doing a good service in giving a larger circulation to facts showing the improved management of an institution which had become a bye-word. *Bedlam!* a term which has been naturalized from its expressing in one word a whole sentence; and what a sentence! "*A Bedlam,*" a hell upon earth, a madhouse, and a loathsome prison combined in one—conjuring up the compound image of murder, madness, and perpetual imprisonment amidst disorder, chains, dark dungeons, and filthy straw. "*Fit for Bedlam,*" qualified for the lowest conceivable depths of degradation and imbecile misery: a prison—where a quarter of a century after Pinel had shown that it was as safe as it was humane to release even the most savage maniacs from chains and dungeons, the parliamentary commissioners discovered that the old horrors were still in fashion; that not one ray of light had broken into the obscure intellects or warmed the cold hearts of the Bedlam Committee; where dozens of human beings whose spirit was not so entirely broken that they could resent no longer cruelty and wrong, were chained night and day by hands and feet to their pallets, naked, except a rug or foul blanket, whilst those who had been reduced by ill-treatment and disease to harmless imbecility were consigned to styes of filthy straw. One "Norris" is mentioned—shrewd and intelligent, but at times furious. Mr. Haslam, the apothecary, recommended a strong cell. The committee decided otherwise. They fixed an upright iron post in the centre of a cell, an iron collar was riveted round Norris's neck, and made to slip up and down this pillar, whilst round his waist was fastened another hoop to which his arms were closely pinioned. He sat on a trough to which his legs were chained. It took twelve years of this misery to wear him out, and visitors who were admitted to stare at him, saw him reading books of biography and history, and found that he could talk sensibly on the topics of the day.

But another quarter of a century has passed, and we have these Reports, out of which neither the most determined grumbler nor the sickliest sentimental admirer of the middle ages could extract one proof of our degeneracy. Quite otherwise. Admitting fully that we are as culpable as a nation as each one is individually, too often knowing the right and following the wrong, yet such evidence as we have now before us is a cheerful proof of the real progress society has made in the path of humanity, and of the indisputable claims which our own art has to the gratitude of mankind. But let us pass on to the Reports themselves.

During 1843, 284 cases were admitted. The number of cures were greater than the preceding year. In 1843, 159 were cured, or 56 per cent. on the admissions, whilst a similar calculation for 1842 shows only 51·31 per cent. In 1844, however, the number of cures again fell short: 286 cases were admitted, and only 128 were discharged cured. A careful examination of the tables explains the reasons of this, and shows that very little faith can be placed in the results of statistical tables unless all the circumstances are taken into consideration. The unfavorable returns of last year lead the reporter into such a minute examination of the circumstances of all the cases as probably would not have been thought necessary, if the results had been favorable. More accurate conclusions, however, are gained from the results of the treatment of many years, and the increased proportion of cures which such tables exhibit is very gratifying. Thus in the middle of the last century the ratio of cures was 33·20 per cent. annually, in 1843 it had reached 56 per cent.: whilst the deaths in the former period were annually *one in every four admissions,* and now they are *one in every seventeen.*

From the advantages resulting from employment, a range of workshops were erected in 1843, that the patients might carry on simple handicraft trades, not with a view to profit, (although a considerable expense is saved even by the patients in whitewashing and painting,) but to withdraw their attention from their own distempered ideas, and to alleviate the monotony of their confinement. The only argument against this plan is the danger which may result from the use of tools. But as it has been found that the patients have been intrusted with hoes and spades in the garden, with carpenter's tools, sledge-hammers and rods of iron at the forge, and "no accident of the most trifling character has occurred," but the patients have been most materially benefited, there were good grounds to infer that the several trades about to be established might be carried on without danger, and with more wide advantage, and it was confidently anticipated that in the course of the spring "an industrious and cheerful colony of artisans would fill these buildings, instead of pacing the galleries in listless idleness and melancholy silence." It is less difficult to employ the women, and for years, the laundry, household work, sewing and knitting have afforded occupation; the present matron has introduced straw-platting, bonnet-making, shirt-making, lace-making, and fancy-work, and the large and cheerful apartment recently complete and furnished as a workroom, affords abundant evidence of the industry of the patients. This institution from its peculiar nature renders profitable occupation difficult. One fourth of the patients only are old and incurable cases, and of course these mere chronic patients can alone be employed with any degree of regularity. The curable patients who form one half of the whole number, cannot remain more than twelve months, and do not on an average remain more than five months.

The Report for 1844 confirms these good accounts.

"The improvements introduced in 1843 now work more smoothly, from greater practice and experience, and are now completely incorporated as part of the system on which the hospital is to be for the future conducted. The workshops have been completed. All the ironwork, painting, plumbing, glazing, carpentering, bricklaying, plastering, and mason's work, engineering, and millwork, now required for the ordinary use of the establishment, is performed in these workshops, thus combining economy with these most important remedial agents, so useful in alleviating the maladies of the inmates." [No accidents have occurred.] "The blacksmith's workshop has been doubled in extent; a large and commodious apartment has been set apart for the engineers, and the carpenters' shops are now nearly twice their former size. In each and all of these are to be seen some of the patients; but their quiet and orderly demeanour, and contented and cheerful industry, render it impossible for a stranger, and even difficult for a governor, to distinguish the patient from his attendant. Several of the patients who were unacquainted with any branch of trade, have been instructed in one or other during their residence; and every advantage is taken of inducing those who can work to occupy themselves, if possible, in those trades. One instance of the kind may be here stated, as it shows the effect of gentle persuasion in this respect. A patient, previously an engineering smith, was in a most desponding state, and it was with great difficulty that he could be prevailed upon to take any exercise. The steward, having at last induced him to visit the engineers' shop, fixed a piece of iron in a vice, placed a file in the patient's hand, and holding his arm, began to use it as if at work. The well-known sound and motion roused the attention of the patient, and the next day he voluntarily began to work, and has continued a constant and industrious workman, and before four months had elapsed he had made and finished a cross head-bar for a steam-engine, with all its necessary joints and sockets ready for immediate use; while the improvement in this patient's bodily health was as great as in his mental disorder; and, like almost all the other patients employed, he expresses his gratitude for it and his sense of its value.

Numerous other instances of a similar nature might be quoted, but to do so is at present unnecessary. All persons conversant with insanity now admit the advantages arising from active bodily occupation in the treatment of the insane; and if any lingering doubts should still remain in the minds of any governor respecting the prudence of the system now pursued at Bethlem Hospital, they must be removed by the knowledge of the important fact, that no accident or misadventure has occurred during the past year in connexion with labour or from the use of the instruments employed. It cannot be denied that the system is carried out with much boldness and decision; and the greatest credit is due to Mr. Nicholls for the vigilant and constant supervision which he maintains over this most essential part of the Institution, as well as to the artisan-attendants, who display the greatest caution and kindness towards those placed under their care and instruction. The absence of accident, and the diminution of restraint, not only justify the system adopted, but advocate its extension wherever practicable.

"The plan is now in complete operation, and the anticipations formed in the last Report of its beneficial effects have been fully realized, not only in promoting the comfort of the patients, but in many instances it has been a main cause of cure, and in all it has tended to alleviate the irksomeness of their confinement, to interest the moody, and divert the energy of the excitable into a course of cheerful industry and congenial occupation." (pp. 4-5.)

Their *amusements* are attended to. There is a library of useful and entertaining books, which is efficiently superintended by an incurable librarian. Chess, draughts, cards, and backgammon have long been allowed with very beneficial effects, and are carried on in a well-furnished and comfortable room appropriated to reading, writing, drawing and other amusements. The committee suggest that a set of maps would be acceptable, as there are many foreigners, and most of the patients come from distant parts of the kingdom.

"In addition to the many obvious advantages arising from a well-considered system of amusements, there is one of great importance, which ought not to be overlooked. The order and content which it produces removes the motive which sometimes was mistaken for the necessity of sending the patients to bed at an early hour. Attendants, however well disposed, if wearied out with vain attempts to soothe and tranquillise patients who, solely for want of occupation, became a cause of perpetual annoyance and vigilance, could hardly be blamed if they formerly looked forward to the hour of removing their troublesome charges to their sleeping-rooms as a welcome release. If this feeling ever existed, it does so no longer. The attendants and nurses join in the games and recreations; thus maintaining the best possible feeling between them and the patients, who now invariably yield to their requests with willingness and alacrity." (p. 47.)

To the women's rooms has been added a piano, which often leads to a dance which is always conducted with gaiety and decorum. By a vote of the general court, permission was given to procure a billiard table which has been done.

"The use of it is granted sparingly and with caution. It is employed chiefly as a means of exercise when the patients are unable to leave the galleries from the state of the weather, or cannot otherwise be induced to exert or amuse themselves. This and the bagatelle boards have fully realized the favorable expectations of the governors."

An instructive case is given. A patient was brought to the hospital so excited that when released from his strait-waistcoat he knocked down three strong men. The next day, although still greatly excited, he was taken into the amusement-room, he appeared astonished at seeing a number of persons reading, playing at cards, draughts, and bagatelle; he became calm, joined in the amusements, remained quite tractable, and was discharged cured.

Such an anecdote is highly instructive. The excitement and violence which seemed to require physical force was at once subdued by the simplest moral means. How apt are we to forget that we have human beings to deal with, not mere pieces of mechanism moved by steam, or galvanism, or gunpowder! Although the complete non-restraint system has not been adopted yet, the governors are not to be considered as opposed to a system, "which though it works well in asylums in which chronic cases and idiots form a large proportion of the inmates, has not been adopted to the same extent in an hospital which receives none but recent cases." Every instance of restraint, however short, is registered, and the diminution, arising mainly from the increased means of employment, during the last five years, has been striking, and attended with the best results. The following is the weekly average of patients under restraint:

| | |
|---|---|
| 1839—11$\frac{33}{52}$ | 1842—3 |
| 1840—13$\frac{20}{52}$ | 1843—3$\frac{4}{52}$. |
| 1841—9 | |

One of the greatest advantages (says the Report) arising from dispensing with restraint, as far as practicable, is the change which it invariably produces in the intercourse between the patients and attendants. They acquire the confidence of the patients, and become the directors of their occupation and the companions of their amusements, instead of exhibiting themselves only in the repulsive character of gaolers.

Each patient has a tepid bath once a week, and a second change of linen. Tea and sugar have been allowed since March last. This costs the hospital £400 a year, but it is a great addition to the comfort of the patients and relieves their friends who could ill afford to supply them. To avoid hurting the feelings of the patients no distinctive dress is required, and knives and forks properly secured have been substituted for the former bone implements; and crockery for the old wooden platters. The table which shows that increase in the number of those who attend the chapel goes hand in hand with diminution of restraint and kinder treatment, is most instructive. In 1839, 27 per cent. attended; in 1843 nearly 49 per cent.: thus within the last five years the number of patients attending chapel have doubled, and within the last ten years have more than trebled! The number of attendants has been increased, and an annual increase of wages allowed, depending on satisfactory conduct. "Too much care cannot be taken in the selection of attendants, and their wages and comforts should be on a liberal scale. The constant trials of their temper and forbearance require qualities of mind and disposition rather than personal strength."

The following passage is too important to be consigned to a Report read by governors only. It goes to the root of the matter, and shows that in the non-restraint system, employment and amusements are essential, and that if lunatics are encouraged to spend their days in listless indolence, they will, like children, as well as all idlers, quarrel with each other, magnify trifles into great things, and become irritable and peevish, and mischievous. It is not only in nurseries that

"Satan finds some mischief still
For idle hands to do."

"Perhaps nothing marks the contrast between lunatic asylums, in which due and ample provision is made for the amusement and occupation of the patients, and those in which, for various reasons, the patients are not employed, than the feeling which exists between the patients and the attendants. In the first, the attendants invariably exhibit a kindness and consideration to the patients which can hardly be looked for in the latter, where, unoccupied and irritable, the patients are a source of continual annoyance and trouble to their attendants and to each other. To suppress confusion, and quell violence, the attendants, in the absence of other means, must have recourse to harshness and severity; their presence is regarded with dislike, and a feeling of continual hostility engendered between them and the patients." (Report, 1843, p. 51.)

The obvious consequences of such idleness is, that restraint will be employed. The irritated keeper will put the riotous ones in handcuffs, muffs, and restraint-chairs, and having rendered them incapable of more mischief, will go back to his game of whist and his beer with his fellows. The connexion between want of occupation and mischief, the metropolitan commissioners have overlooked in their view of the non-restraint system, and among the arguments given in favour of it they have entirely omitted, as we have before stated, one of the strongest "that mechanical restraint is an exciting cause of suicidal propensities,"—and which is thus proved. Eighty-one patients or more than 28 per cent. of those admitted were reported as having suicidal tendencies, and thirty-seven of these or 13 per cent. had attempted suicide previous to admission, and yet although only three patients have been on an average under restraint every week, there has been no attempt at suicide during the last year! "No stronger evidence (the Report continues) can be given of the tendency of mechanical restraint to excite suicidal attempts than that supplied from the records of the hospital, from which it appears that during the twenty years, from 1750 to 1770, *when every patient was under restraint*, the suicides were in the proportion of 1 to 202; whereas, during the last twenty years, the proportion has been only 1 in 963." This evidence is invaluable. It gives us the highest satisfaction to be the means of diffusing so conclusive, so unanswerable, so deeply important a fact. A medical proprietor of a licensed house has recently given as one of the reasons for restraints, the responsibility he is under for the lives of his pauper patients. If they kill themselves he loses his character, and as he supposes, he prevents this evil by hand-chains; but such a fact as this must alter his opinion, as it proves that his supposed remedy is the most ready means to produce the propensity he fears.

The Report for 1844 adds still further evidence in favour of the non-restraint system:

"It is deemed expedient," says the Report *from Bedlam!* "*rather than impracticable*, to adopt the principle of dispensing wholly with restraint under all circumstances; yet every opportunity is taken of confining it within the narrowest possible limits. Personal restraint has been reduced to one-tenth of what it was six years ago; and it is most gratifying to be able to state, that it has been reduced during this year to one half of what it was in 1843. The following returns show the average weekly number of patients in restraint during the last six years:—

| | |
|---|---|
| 1839—$11\frac{33}{52}$ | 1842—3 |
| 1840—$13\frac{20}{52}$ | 1843—$3\frac{4}{52}$ |
| 1841— 9 | 1844—$1\frac{35}{52}$." (p. 49.) |

—Thirty-six per cent. of those admitted this year were reported as having attempted or as being predisposed to commit suicide, but no suicide has happened in the curable wards this year.

"Every year" adds the Report, "confirms the view that restraint is a highly-exciting cause of suicide; and the fact that no untoward circumstance has occurred in Bethlem, with so large a number of dangerous patients, while mechanical restraint has not been used for two patients a week, is a most striking illustration of the advantage of this system, and the Committee have the authority of the resident officers to state that nothing has occurred to shake their confidence in the advantages of the system which they superintend.

"It deserves consideration, whether it might not be of advantage in the treatment of suicidal patients, to construct a sleeping-room in each gallery, to contain four or six beds, which might easily be effected by throwing two or three sleeping apartments into one. In addition to securing extended accommodation, the companionship thus afforded would be beneficial, as well in assuring the timid patients as in deterring suicidal patients from any attempts against themselves. Suicide is seldom committed in the presence of another, while the tendency may be excited by the loneliness of a separate sleeping apartment. This view is confirmed by the practice in other well-regulated asylums for the insane." (pp. 50-1.)

*Medical pupils.* The governors have with much judgment increased the facilities for making Bethlem a school for the instruction of pupils in the practical knowledge of insanity. Each physician may take pupils at 15 guineas for six months, and 20 guineas for twelve months, the number not limited, but not more than four to accompany him when he makes his visits. In addition to this the governors have authorised the committee to nominate two pupils, one from St. Bartholomew's and one from St. Thomas's Hospital, to attend the physicians' practice at Bethlem; the fees to be paid to the physicians out of the funds of the hospital. As each pupil will remain six months, four pupils will thus attend annually; and they are to be selected "not only as a reward for their assiduity and proficiency in the study of their profession, but also for their general propriety of conduct, to be certified by the medical officers of their hospital, in addition to the recommendation from the president and treasurer on behalf of the governors." Each of these pupils is expected to perpare, and present at the end of his term, an essay on the nature and treatment of insanity, to the governors of Bethlem Hospital; a condition of which we rather doubt the prudence or the useful results.

This we have before urged, and the committee allude to our arguments that to France we are indebted for our best works on insanity, for in that country every insane hospital has a class of students attached to it. On other grounds this will be a great boon. The young practitioner of medicine leaves his studies and commences practice without probably having attended one case of lunacy: and to his care falls the treatment of pauper lunatics at the earliest stage, when wrong treatment is often productive of irremediable mischief. His head is full of pathological anatomy, and antiphlogistic theories. The symptoms of determination of blood to the head which are well marked, are supposed to require the same treatment as active inflammation. The patient is roughly purged "to clear him out," not with a view to improve his secretions; the lancet is thrust into his arm, and he is handcuffed in a strait-waistcoat with brute violence,—when the application of cold to the head would probably have been sufficient, in a class of patients generally much below par, to have checked or restrained the determination of blood, whilst attention to the improvement of the secretions by aperients and alteratives, and a nutritious unstimulating diet with the judicious use of morphia (in some cases), and quiet, would have been the right practice. And what does wrong practice in this early stage end in? Bleeding the exhausted body of a half-starved pauper, and irritating by coercion and violence his exquisitely sensitive brain, are precisely the means best adapted to render him one of that large class termed incurables, with which our county asylums and workhouses are yearly more incumbered. Sounder views will best be promulgated by actual experience, and although but a few pupils can benefit by Bethlem yet every instructed man is a light by which many are illuminated.

The following case given in the Report for 1844 is a specimen of what is we fear very common:

"The first case alluded to was that of a male patient brought for admission in a very violent and excited state, having, in addition to a strait-waistcoat, his arms bound with cords, his wrists secured by a belt, and his legs confined with strong webbing. In extenuation of such severe measures, his relative, who accompanied him, assured the steward that this restraint was absolutely necessary, 'as he was very difficult to manage, and that it had even required as many as six men to place him under coercion.' The first thing done on admission, was to release the patient from all restraint, and although, as might be expected, he remained for some days in a highly excited state, so as to require the constant watching of one, and sometimes two attendants, no personal coercion was afterwards used during the whole time he remained under treatment. In a few days the symptoms of an inflammatory affection of the chest appeared, from the effects of which, combined with great cerebral excitement, he died, in a fortnight after admission. A post-mortem examination of the body proved that the breast-bone and one rib were fractured; the interior of the chest was also found much affected in consequence of the irritation which the broken bones produced on the lining membrane, and it can hardly be doubted that these severe injuries occurred in the struggles which took place when so much restraint was imposed." (pp. 39-40.)

The first Report refers with gratification to the increased proportion of a class above that of paupers which have been admitted during 1843, and the Report for 1844 dwells on the same topic.

"The table indicating the occupations of the patient will be read with some interest. Amongst the males are included artists, chemists, clerks, clergymen, military officers, students, and schoolmasters; and in the list of females will be found several dress-makers, embroiderers, four gentlewomen, lodging-house keepers, officers' widows, and the wives and daughters of many respectable tradesmen. These are persons with peculiar claims to our sympathy; the rich in their afflictions can command all the aids and comforts which wealth can purchase; the pauper lunatic finds within the walls of a county asylum that care and sympathy to which he may long have been a stranger; but when this fearful calamity falls upon one dependent on his own exertions to maintain a respectable position in society, the consequences are, indeed, distressing; his scanty funds are rapidly absorbed, his sources of income necessarily fail; and his family, after all their willing, but fruitless sacrifices, are borne down to poverty by the overwhelming calamity which has prostrated their protector. Until, therefore, some asylum is instituted, in which this class of patients, so deserving of the generous sympathy of the public, can be received, the liberal policy of the governors who have admitted such patients as those enumerated, and provided them with accommodation and general treatment suitable to their feelings and former position, cannot be sufficiently applauded. The library and workrooms, with the personal comforts arising from the stated use of the tepid bath; the substitution of crockery-ware at meals; the more frequent changes of body linen; and the improved appearance of the bedding materials,—are not only fully appreciated by the patients, but have removed many causes of irritation which operated injuriously upon them, by making too strong a contrast with their habits of life before admission. Every opportunity is taken of assimilating the treatment, as far as practicable, to the previous habits of the patients, the propriety of which is most obvious." (59-60.)

That more real charity is displayed in giving the temporary succour of the hospital to this class, unprovided for in our county asylums or workhouses, all must agree with the committee. And it is with the liveliest satisfaction that we are now enabled to announce that the claims and necessities of this large class, are about to be met by the establishment of an institution for their especial benefit. The subject of such an asylum had engaged the consideration of Dr. Conolly and several of his friends, for the last two years; but it is only recently that steps have been taken for its actual institution. At a public meeting held at the Freemasons' Hall, on the 10th July, composed of many benevolent members of the medical profession and of other philanthropic persons, and presided over by that great redresser of wrong in all its forms, Lord Ashley, the "Asylum for the Insane of the Middle Classes of Society," took its name among the charities which do honour to this country, and will, we trust, ere long, have as its local habitation one, not the least conspicuous, of the architectural structures that adorn the vicinity of the metropolis. In this institution, for the moderate sum of 25 or 30 shillings per head per week, all the advantages and resources enjoyed in the best institutions for the treatment of this disease, will be ensured for a class above those which would be admitted into Bethlem, but still not wealthy enough to remunerate the proprietors of smaller asylums. "Persons of good family and of small income, officers on half-pay without fortune, clerks in the public service, or in offices of various descriptions, with salaries dependent on their continued exertions, tradesmen of small property, or retired with a scanty independence, clergymen with small incomes, men of the other learned professions who have no other means of support, tutors, governesses, and many others whose position does not require to be specified, but who in ordinary circumstances enjoy all the comforts of respectable life, are at once involved in pecuniary embarrassment, and often eventually reduced to absolute poverty and want, by the occurrence of insanity in themselves, or in the person of a child, a wife, a parent, or some dependent relative."* It is proposed to raise, by donations, the sum of £30,000, which is considered, after the most careful calculations, to be sufficient to purchase 25 acres of freehold land near London, and to erect an asylum capable of accommodating from 250 to 300 patients. This house will, of course, by its construction and organization, be capable of supplying the inmates with all those occupations and amusements, those means of moral, intellectual and physical improvement which tend to the recovery of those who are curable, and to the solace, comfort, and amendment of all. These objects could be provided at a moderate expense in a large asylum only, and it is fortunate that the size of an asylum rather facilitates than obstructs the attainment of these advantages, by rendering classification more easy and thereby securing tranquillity to some, and to the majority that cheerfulness

* Prospectus of the Asylum for the Insane of the Middle Classes.

which results from a variety of objects which excite attention, and interest, and stimulate to exertion and imitation, the want of which renders smaller establishments oppressive by their dullness, and monotony. It is further proposed, as an integral part of the plan, that a charitable fund shall be raised by donations, subscriptions, and bequests, the interest of which shall be applied for the benefit of the more distressed among those for whose relief the asylum is destined, a certain number of whom are to be elected from time to time from those patients who have been a year in the asylum, and placed on the list of patients received for a lower payment, say for fifteen or ten shillings per week, or even without any payment whatever. By this means timely support will be afforded to families who have made great exertions to furnish the annual payments for a time, but are unable to continue them, and to cases where the occurrence of insanity has wholly deprived the sufferers and their families of the means of support, and where the assistance of friends can only be relied on for a limited period. Those who are most conversant with the wants of society are, it is believed, unanimously of opinion that such an institution as this is urgently needed, and that it would be of important service in relieving the misery of those on whom this worst disease falls heaviest; those who have been reared in comfort and with a share of the refinements of life, but who by the same blow which renders these comforts necessary, are deprived of the means of procuring them.

But to return to these Reports of Bethlem. Fourteen new wards are erecting for the exclusive use of convalescents. The best authorities in insanity, (Esquirol, Pasquier, and others,) insist on the necessity of the complete separation of the convalescent from those at a stage less advanced, and to these wards no patients are to be admitted who are not reported cured, for the purpose of preparing them gradually for the entire control over their actions when finally discharged. The records of the hospital prove that many readmissions have arisen from the sudden change of scene which patients have experienced from want of such a probationary period.

The Report of last year confirms the opinion that the non-restraint system keeps up confidence and good feeling between the attendants and patients. The difficulty of obtaining good attendants, which is the greatest difficulty to be overcome in lunatic asylums, has been partly overcome in Bethlem. Attached to the hospital and forming part of it is the house of occupations to which neglected and destitute children are sent, as some of the best conducted have been trained during the last year to act as attendants and nurses for the insane patients, in addition to learning trades and the duties of domestic servants.

> "No less than twelve boys are constantly employed with the convalescent patients in the workshops of the hospital, under the tradesmen-assistants, with great advantage to both parties. The convalescent associates with sane persons, no slight aid to ultimate recovery, while the boys familiarized with the aspect of lunacy, learn forbearance, kindness, and a respect for the feelings of others, which operate beneficially upon their own feelings and conduct, while their experience, which few can obtain, may be of substantial value to them in after life."

This experiment of employing boys as attendants, which has only been tried one year, is of great interest. Orphan and destitute children form so numerous a class in our union workhouses, that much mutual benefit might arise from their employment in this way in county asylums. The union workhouses would be relieved and the asylum benefited, whilst the public expense would not be increased.

These Reports furnish strong evidence in favour of county asylums, as they show how curable recent insanity is, and how greatly its evils may be relieved by such classification, employments, and amusements, as alone can be methodically pursued in a large institution. Every day we are more convinced of the necessity for county asylums throughout the kingdom, to give the poor the best chance of cure, so as to reduce, or at least not to increase that class who are condemned to perpetual imprisonment and uselessness, a burden to themselves and to the public; and Lord Ashley's new Lunatic Act is one of those large and liberal provisions which, we trust, will prove as beneficial to the insane poor as it is worthy of the benevolence of its author, and the beneficence of this great country.

The criminal department of Bethlem contains ninety-two inmates, seventy-three men and nineteen women. This is virtually a government prison, the duties of the governors being confined to the safe custody of the inmates and the superintendence of the internal economy. The patients "are treated in the same careful and gentle spirit which pervades the other departments."

In closing our extracts it would be injustice not to mention the names of Mrs. Hunter, the matron; and of Mr. Nicholls, the steward, who superintend the male and female departments. The well-conceived plans and skill with which the former carries them into effect, and the readiness in resources, the general ability and masterly skill of the latter are spoken of in high terms of well-deserved eulogy by the committee. The author of this excellent Report conceals his name, and though we believe him to be a much-respected member of our own profession, we do not think proper to strip him of the incognito he has chosen to assume. Of this, however, he may be assured, that his labours in ameliorating the condition of the inmates of Bethlem have the highest reward—the blessings of those who have benefited by them. Presidents and committees, according to our experience, often get public credit for the labour of one or two who wish no higher reward than the mere gratification of doing good—the living powers of the establishment, working noiselessly and unseen.

> "Sagt! was füllet das Zimmer mit Wohlgerüchen? Reseda,
> Farblos, ohne Gestalt, stilles bescheidenes Kraut." (Goethe.)

Glad were we to hear lately from one of our sight-seeing friends that he had just been over "Bedlam," but that there was really nothing to see: he should not have known that it had been a madhouse. The attendant, too, he said, (most rightly and judiciously we thought) hurried the whole party through the rooms as if it were a duty he did not like, and wished to get over quickly.

"Heureux le peuple dont l'histoire ennuie;" and happy Bedlam when it is no "*spectacle*," when there is nothing excitingly shocking to tell about. We turned to the description of a keeper sixty years since, who, when the ladies whom he was conducting through Bedlam, were shocked with the clinking of the chains, the wildness of the cries and imprecations, and begged to return; "he seemed surprised at their uneasiness, and was with difficulty prevailed on to leave that part of the house without showing them some others; who, as he expressed it, in the phrase of those that keep wild beasts for show, were much better worth seeing than any they had passed, being ten times more fierce and unmanageable."

But why is Bethlem a London sight at all? Surely there are sights enough—Zoological Gardens, Fancy Fairs, General Tom Thumb, Mesmeric Mountebanks, Exeter Hall, and others innumerable. There is every excuse too now for shutting it up. The gentle public will not even be deprived of one of its excitements, its pleasing horrors; for humanity has spoiled its piquancy, and made a visit which afforded reminiscences for life, flat, stale, and unprofitable. Such tidings as these Reports bring, make us rejoice that a name recalling the best associations of which our highest nature is capable of delighting in, but which had been so corrupted and disfigured as to bear so faint a resemblance to its original, is once more restored to our everyday tongue: "BEDLAM" is no more; and for the future "BETHLEM," whilst it expresses the original idea of the hospital, will convey the right impression of its actual condition.

---

